# Altered epithelial barrier functions in the colon of patients with spina bifida

**DOI:** 10.1038/s41598-022-11289-3

**Published:** 2022-05-03

**Authors:** Charlène Brochard, Guillaume Bouguen, Raphael Olivier, Tony Durand, Sébastien Henno, Benoît Peyronnet, Mael Pagenault, Chloé Lefèvre, Gaëlle Boudry, Mikael Croyal, Alain Fautrel, Maxime Esvan, Alain Ropert, Anne Dariel, Laurent Siproudhis, Michel Neunlist

**Affiliations:** 1 Service d’Explorations Fonctionnelles Digestives, CHRU Pontchaillou, Université de Rennes 1, 2 rue Henri le Guillou, 35033 Rennes Cedex, France; 2 The Enteric Nervous System in Gut and Brain Disorders INSERM, TENS, Université de Nantes, Nantes, France; 3 Inserm, CIC 1414 (Centre d’Investigation Clinique de Rennes), CHU Rennes, 35000 Rennes, France; 4 Centre Référence Maladies Rares Spina Bifida, CHRU Pontchaillou, Rennes, France; 5 Service des Maladies de l’Appareil Digestif, CHRU Pontchaillou, Université de Rennes 1, Rennes, France; 6 Institut Numecan, INSERM, INRAE, Univ Rennes, Rennes, France; 7 Service d’Anatomopathologie, CHRU Pontchaillou, Rennes, France; 8 Service d’Urologie, CHRU Pontchaillou, Rennes, France; 9 Université de Nantes, CHU Nantes, INSERM, CNRS, SFR Santé, Inserm UMS 016, CNRS UMS 3556, 44000 Nantes, France; 10 CRNH-Ouest Mass Spectrometry Core Facility, 44000 Nantes, France; 11 Plateforme H2P2, Université de Rennes, Rennes, France; 12 Service de Chirurgie Pédiatrique, CHU Marseille, Marseille, France

**Keywords:** Physiology, Gastroenterology, Medical research

## Abstract

Our objectives were to better characterize the colorectal function of patients with Spina Bifida (SB). Patients with SB and healthy volunteers (HVs) completed prospectively a standardized questionnaire, clinical evaluation, rectal barostat, colonoscopy with biopsies and faecal collection. The data from 36 adults with SB (age: 38.8 [34.1–47.2]) were compared with those of 16 HVs (age: 39.0 [31.0–46.5]). Compared to HVs, rectal compliance was lower in patients with SB (p = 0.01), whereas rectal tone was higher (p = 0.0015). Ex vivo paracellular permeability was increased in patients with SB (p = 0.0008) and inversely correlated with rectal compliance (r = − 0.563, p = 0.002). The expression of key tight junction proteins and inflammatory markers was comparable between SB and HVs, except for an increase in Claudin-1 immunoreactivity (p = 0.04) in SB compared to HVs. TGFβ1 and GDNF mRNAs were expressed at higher levels in patients with SB (p = 0.02 and p = 0.008). The levels of acetate, propionate and butyrate in faecal samples were reduced (p = 0.04, p = 0.01, and p = 0.02, respectively). Our findings provide evidence that anorectal and epithelial functions are altered in patients with SB. The alterations in these key functions might represent new therapeutic targets, in particular using microbiota-derived approaches.

**Clinical Trials:** NCT02440984 and NCT03054415.

## Introduction

Anorectal disorders, including faecal incontinence (FI) and constipation, are common in patients with spina bifida (SB)^1–3^ and affect their quality of life^4^. The pathophysiological basis of the symptoms experienced by patients with SB has not been clearly established, contributing to inadequate care^5^. We showed the absence of an association between the neurological level and severe FI^3^, and FI in patients with SB is mainly associated with rectal abnormalities^6^. Using a model of rectal isobaric distensions with an electronic barostat is the gold standard to study rectal functions^7–10^. However, an analysis of rectal functions using a barostat has never been conducted in patients with SB. Therefore, this model should be used to better characterize the rectal function of patients with SB and to obtain novel knowledge of other key rectal functions, such as epithelial barrier functions.

The intestinal epithelium lining forms a functional barrier between the host and the contents of the intestinal lumen^11^. It prevents the passage of noxious contents while allowing the absorption of nutrients and electrolytes^11^. Crossing of this barrier occurs via two routes, either between epithelial cells via the paracellular pathway or through epithelial cells via the transcellular pathway. Among the most important structures of the intestinal barrier are epithelial tight junctions (TJs), which regulate paracellular permeability^12^. They are formed by transmembrane proteins such as claudins and occludins connected to the actin cytoskeleton via high molecular weight proteins such as zona occludens (ZO-1, ZO-2 and ZO-3)^12^. Impairment of intestinal epithelial barrier (IEB) functions is often associated with altered expression of key TJ proteins^13,14^ and leads to an increase in paracellular and/or transcellular permeability^15,16^. Increased permeability of the IEB along with changes in TJ protein expression have been consistently reported in several digestive disorders, such as inflammatory bowel diseases^17,18^ and irritable bowel syndrome (IBS)^13,14^, or even in neurological diseases, such as Parkinson’s disease^19^. Various factors have been shown to contribute to altered barrier functions, in particular inflammatory mediators such as cytokines (TNFα, IFNγ or TGFβ1), mast cell mediators such as proteases^20^ or even neurotrophic factors such as glial cell line-derived neurotrophic factor (GDNF)^21^. In addition to known host-derived mediators, increasing evidence has identified the gut microbiota and their metabolites as additional factors contributing to the maintenance of IEB functions. In particular, short-chain fatty acids (SCFAs), such as butyrate or acetate, reinforce colonic barrier functions either directly by modulating TJ proteins expression or indirectly by regulating inflammatory processes^22^. However, the levels of inflammatory mediators and SCFAs in patients with SB currently remain unknown, as well as the levels of other cytokines and/or TJ proteins.

The aim of this study was to improve our knowledge of the pathophysiology of anorectal disorders in adults with SB compared with healthy volunteers (HVs) by studying (1) anorectal physiology using a model of rectal isobaric distensions with an electronic barostat, (2) the function and structure of the IEB, (3) the inflammatory state of the IEB from colonic biopsies and (4) factors contributing to the maintenance of IEB functions and levels of SCFAs.

## Subjects and methods

### Study approval

The study protocols were approved by the local Committee on Ethics and Human Research (Comité de Protection des Personnes) (*Anospin* = N° IDCRB 2015-A00082-47; N° ANSM 150138B-42; N° CPP 15.03.12 and *Anosain* = N° IDCRB 2016-A01541-50; N° ANSM 2017020800081; N° CPP 17.02) and registered at ClinicalTrials.gov (identifiers *Anospin* = NCT02440984; *Anosain* = NCT03054415). Written informed consent was obtained from each patient and from each HV according to the principles of the Declaration of Helsinki.

### Population with spina bifida and clinical evaluation

Adult patients with SB consulting at the “*Centre de Référence Spina Bifida-Dysraphismes”* in France were included prospectively and consecutively since June 2015. As previously described^3,6^, the main characteristics recorded were sex, age, height, weight, type of SB (open/closed), neurological level, ambulatory status and ventriculoperitoneal shunt. The questionnaire focused on the main anorectal complaints (past history of faecal impaction, dyschezia, mucus discharge, anal bleeding, Bristol stool^23^, number of stools/week, abdominal pain, and self-reported faecal and urinary incontinence). Anal digitation to defecate, use of softeners, antidiarrhoeal drugs, and anticholinergic drugs were recorded. A physical exam was performed, and the following data were recorded: gaping anus, resting tone of the anal canal (hypertonia, hypotonia, and normal tone), anal canal sensitivity, and perianal sensitivity (pin prick/light touch).

Constipation was assessed using the validated Knowles-Eccersley-Scott Symptom Constipation Score (KESS 0–45)^24^. Constipation was defined as a KESS score ≥ 10. Faecal continence was assessed with the Cleveland Clinic Incontinence Score (CCIS 0–20)^25^, and bowel dysfunction was assessed with the validated neurogenic bowel dysfunction score (NBD 0–47)^26^. We chose to define incontinence severity based on the FIQL^27^. Three classes of clinical severity for FI were defined according to the study by Rothbarth et al.^28^: Cleveland Clinic classification scores of 0–8, 9–12, and 13–16. FI was defined as a CCIS ≥ 5, and severe incontinence was defined as a CCIS ≥ 9. Moderate bowel dysfunction was defined as an NBD score ≥ 10 and ≤ 14, and severe bowel dysfunction was defined as an NBD score ≥ 14^26^. Soft stools were defined as a Bristol stool score of 3 or 4^23^. Each patient included underwent anorectal manometry with a rectal barostat and a short colonoscopy in the same session.

### Healthy volunteer population and clinical evaluation

Sixteen HVs who were comparable in age and sex to patients with SB were included. The following exclusion criteria were used: pregnant or breastfeeding women; anticoagulation curative treatment or known haemostasis disorders; history of anal or rectal surgery, ileoanal anastomosis; Crohn's disease or ulcerative colitis; irritable bowel syndrome defined according to the ROME III criteria; history of pelvic radiotherapy, rectal or anal neoplasia; anorectal symptoms including constipation, FI, and anorectal pain; use of a retrograde or antegrade colonic irrigation system; regular use of laxatives; microscopic abnormality of the colonic mucosa as collagen colitis or colitis microscopic; exclusion period mentioned on the national file of persons who participate in biomedical research; safeguard of justice, guardianship and tutorship; and private persons of freedom. All subjects were deemed to be healthy based on a complete medical checkup, including interviews, physical examinations, and proctoscopic examinations of the rectum and anal canal. Each HV included underwent anorectal manometry with a rectal barostat and a short colonoscopy with biopsies in the same session.

### Anorectal manometry and rectal barostat

Anorectal manometry with an electronic barostat was performed after an enema with 500 ml of warm water on the day of the short colonoscopy. Anorectal manometry using an electronic barostat was performed. Each subject was placed in the left lateral position on a padded table in a quiet environment. The bag and the manometric device were lubricated and inserted into the rectum through an anal proctoscope such that the distal attachment site was 5 cm from the anal verge or immediately above the puborectalis muscle. The device had distinct markings that remained visible to the investigator throughout the duration of the experiment.

Anal canal pressures were monitored using a three-lumen water-perfused catheter assembly (R3B, Mui Scientific, Mississauga, Ontario, Canada) with radially distributed side holes to record the mean maximal resting pressures in the upper and lower anal canal and anal canal length. Each of the three lumens was perfused with distilled water at a rate of 0.1 ml/min from an electrically powered compressed pneumohydraulic perfusion system (PIP4-4, Mui Scientific, Mississauga, Ontario, Canada). The mean squeeze pressure in the lower anal canal was obtained during a 30-s squeeze. Abdominal pressure and dyssynergic defecation were recorded during the effort. Abdominal pressure was reflected by rectal pressure. The rectal perception thresholds and rectoanal inhibitory reflex (RAIR) were recorded using isovolumic distension with latex balloon air inflation in individuals without a latex allergy. The rectal perception thresholds were defined as follows: threshold volume for first constant sensation, defecatory urge volume, and maximum tolerable volume (MTV). The RAIR was defined as present if the amplitude reduction was at least 25% of the resting anal pressure. Amplitude reduction was measured from the resting pressure to the lowest point of the RAIR. The percentage of amplitude reduction was calculated with the resting pressure set to 100%^10^.

Rectal distension was performed by placing a highly compliant polyethylene bag within the rectum and connecting it to the electronic barostat (ABS, Saint Dié, France), as previously described^9,29^. The polyethylene bag had a maximum capacity of 800–850 ml. The maximum rate of air flow was 60 ml/s. The barostat maintained a constant preselected pressure within the rectal bag through an electronic feedback mechanism. When the rectum accommodated or relaxed its content, air was injected, and the bag volume increased. In the present study, phasic distensions were performed by rapidly inflating the bag to successive predetermined ascending levels of pressure (increment of 5 mmHg). Each level was maintained for 60 s and separated from the next by a 60 s rest period at 0 mmHg. Pressure limits were identical during the entire study (1–31 mmHg). In response to rectal distension, the following parameters were monitored or derived from the recorded data:

- Anal pressure in both the upper and lower parts of the canal anal. The upper part is conventionally thought to reflect the manometric activity of the internal anal sphincter and the lower part reflects the activity of the external anal sphincter. At each pressure step, anal pressure was defined as the mean pressure recorded at the level investigated
- The rectal anal inhibitory reflex (RAIR) was defined as the residual pressure measured in the upper part of the anal canal for each step of rectal distension. Maximal rectal pressure was recorded during each phasic distension to analyse the occurrence of rectal contraction.
- Maximal rectal volume recorded at the end of each step of preselected pressure
- Rectal compliance (initial volume) is defined as a change in volume in response to increasing pressure. It represents the difference between the volume at resting state and the volume measured when the preselected pressure was just reached. Because this variation in volume is calculated as the variation between two levels of pressure, it is presumed to reflect rectal compliance. Each volume variation was then plotted against the pressure to produce a pressure–volume (*P*–*V*) curve. “Global compliance” was measured as the gradient of the steep linear aspect of the curve. “Global compliance” was used for the correlation studies.
- Rectal tone, which is defined as the volume variation at constant rectal pressure, is the variation in volume in response to a stabilized preselected pressure (maximal volume–initial volume). A smaller change in volume corresponds to an increase in rectal tone, and a larger change in volume corresponds to a decrease in rectal tone.
- Sensation intensity at each pressure step was measured using a 6-point Likert scale ranging from 0 (no sensation) to 6 (intolerable pain).

### Collection of colonic biopsies and faecal samples

Subjects provided fresh faecal samples collected in the morning on the day of the colonoscopy. The faecal samples were then aliquoted and snap frozen in liquid nitrogen at the time of collection and stored at − 80 °C until analysis. A left colonoscopy was performed for each subject after cleansing of the distal colon with two 500-ml water enemas performed before the procedure. Biopsies (n = 14/subject) were obtained from the left colon during the colonoscopy. For all participants, two mucosal biopsies were obtained for routine haematoxylin and eosin histology (HES) to exclude the presence of microscopic colitis. The remaining 12 biopsies were used for the analyses described below.

### IEB changes studies

The 12 mucosal biopsies and the faecal samples were used to study the IEB. All methods used to study IEB are listed in Table 1. The methods are detailed in Supplementary material 1.

**Table 1:** Summary of the methods used for the analysis of biopsies and stool samples.

		Methods
IEB function	Ex vivo assessment of colonic para- and transcellular permeability	Ussing chambers
IEB morphology	Expression levels of tight junction proteins (occludin, Claudin-1, ZO1 and cingulin)	Western Blot
	Expression of tight junction proteins (Claudin-1, ZO1, JAMA) Proportion of KI67+ cells	Immunohistochemical studies
	Collagen proportion (Sirius red staining)	Staining studies
	Number of crypts/sample, mean area of a crypt, total crypt density, total density of crypts (%), elliptic form of crypts (major axis on minor axis), mean circularity, mean roundness, collagen total area, and collagen % area	Full-field optical coherence tomography
Regulation of IEB remodeling	mRNA expression of GDNF, TIMP, MMP1, and MMP2	qPCR
Inflammation of IEB	mRNA expression of TGFβ1, IL8, IL6, TNFα, IFNγ,	qPCR
IEB environment	Concentrations of BAs and short-chain fatty acids in stools	Liquid and gas chromatography–mass spectrometry

*IEB* intestinal epithelial barrier, *qPCR* quantitative polymerase chain reaction, *BA* bile acids, *SCFAs* short fatt acids.

### Statistical analysis

Categorical variables are reported as numbers and percentages; patients with SB and healthy volunteers were compared using Chi^2^ tests or Fisher’s tests. Continuous variables are presented as medians and interquartile ranges and were compared using Mann–Whitney Wilcoxon tests. Pearson’s correlation coefficients were calculated and Fisher’s tests were performed to verify whether Pearson’s correlation coefficients were significantly different from 0. For each type of distension, two-way (group, pressure step), repeated measures ANCOVAs were performed on anal pressures, rectal volumes, and perception scores. Variables that significantly differed between patients with SB and HVs at inclusion were also included in the models. Statistical analyses were performed using SAS software, Version 9.4 (SAS Institute Inc., Cary, NC, USA). All statistical tests had a significance level of 0.05.

## Results

### Study population and healthy volunteers

The characteristics of the 36 patients with SB are listed in Table 2 and were compared with those of the 16 HVs. The two groups did not differ in age (SB: 38.8 [34.1–47.2], HV: 39.0 [31.0–46.5] years; p = 0.9869), sex ratio (SB: 19 men (52.8%), HV: 9 men (56.3%); p = 0.8167) or BMI (SB: 25.6 [21.8–29.9], HV: 23.4 [20.3–26.7] kg/m^2^; p = 0.2116). Among the patients with SB, open spinal dysraphism and lumbar neurological level predominated, and one-quarter were obese. Self-reported FI was reported by 24/36 (66.7%) patients, and severe FI was reported by 18/36 (50.0%). Constipation (KESS score ≥ 10) was predominant (28/36; 77.8%) in patients with SB. One-third had severe bowel dysfunction. Overall, 3/36 (8.3%) were taking anticholinergic drugs, 5/36 patients (13.9%) had an ileal conduit, 4/36 (11.1%) had a cystostomy, 14/36 (38.9%) used intermittent catheterization and 12/36 (33.3%) voided spontaneously. Three patients (8.3%) had severe chronic kidney disease. Overall, considering the GIQLI score (0–144), patients with SB had a significantly impaired quality of life (SB: 88.0 [75.0–104.0], HV: 138 [134.5–141.5]; p < 0.0001).

**Table 2:** Characteristics of patients with spina bifida.

Variable	*N(%) or* median [IQR] N = 36
Age (years)	38.8 [34.1–47.2]
Male sex	19 (52.8)
Parity	6 (35.3)
BMI (kg/m^2^)	25.6 [21.8–29.9]
Obesity (BMI > 30 kg/m^2^)	9 (25.0)
Open spinal dysraphism	23 (63.9)
Neurological level	
Thoracic	2 (5.6)
Lumbar	23 (63.9)
Sacral	11 (30.6)
Able to walk	30 (83.3)
Shunt valve	8 (22.2)
**Digestive characteristics**	
Past history of faecal impaction	12 (33.3)
Dyschezia	30 (83.3)
Mucus discharge	13 (36.1)
Anal bleeding	19 (52.8)
Loose stool (vs. no loose stool)	6 (16.7)
Number of stool/week	3 [2.0–5.0]
Abdominal pain	26 (72.2)
Anal digitation to defecate	17 (47.2)
Use of softeners/laxatives	13 (36.1)
Use of anti-diarrhoeal agents	3 (8.3)
Gaping anus	1 (2.8)
Anaesthesia/hypoesthesia perianal	28 (77.8)
KESS score	16 [10.0–20.5]
KESS score ≥ 10	28 (77.8)
CCIS	9 [3.0–13.0]
CCIS ≥ 5	23 (63.9)
CCIS ≥ 9	18 (50.0)
NBD score	11 [7.0–15.5]
10 ≤ NBD ≤ 14	8 (22.2)
NBD ≥ 14	12 (33.3)

*BMI* body mass index, *CCIS* Cleveland Clinic Incontinence Score, *KESS* Knowles-Eccersley-Scott Symptom Constipation Score, *NBD* neurogenic bowel dysfunction, *IQR* interquartile range 25% and 75%.

The anorectal characteristics of the patients with SB were compared with those of the HVs (Supplementary material 2). In the population of patients with SB, 22/36 (61.1%) had no anal contraction. Overall, 7/36 (19.4%) patients had a latex allergy, and rectal perception was not evaluable in these patients during anorectal manometry. Among patients with SB without a latex allergy, 2/36 (5.6%) had MTV > 330 mL. No HVs had MTV > 330 mL. The RAIR percentage of relaxation amplitude and duration were significantly greater in patients with SB.

### Anal response, rectal adaptation and rectal perception to rectal isobaric distensions

**Figure 1:**
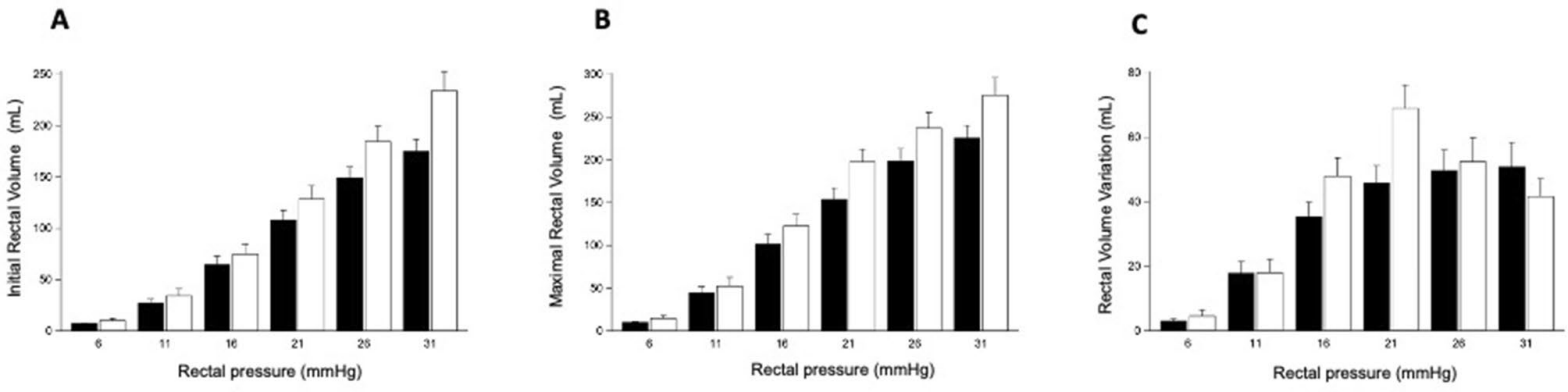
Rectal adaptation to isobaric distension. (**A**) Increasing the pressure of distension increased the recorded initial rectal volume (pressure effect, p < 0.0001). Compared with the HVs, the recorded initial rectal volumes (rectal compliance) were significantly lower in the patients with SB (group effect, p = 0.01; interaction group pression, p = 0.04). (**B**) Increasing the pressure of distension increased the recorded maximal rectal volumes (pressure effect, p < 0.0001). (**C**) Increasing pressure of distension decreased the rectal tone (maximal volume–initial volume) up to the treshold of 21 mmHg (pressure effect, p < 0.0001) for both groups. Compared with the HVs, rectal tone was significantly higher in the patients with SB up to the treshold of 21 mmHg (group effect, p = 0.08; interaction group pression; p = 0.001). *Patients with Spina Bifida; O Healthy volunteers.

The pressures recorded in the upper part of the anal canal as a function of induced rectal pressures were comparable with those recorded in the lower part of the anal canal (Supplementary material 3). Rectal distensions induced the pressure-dependent relaxation of the anal canal (pressure effect; *p* < 0.0001). Anal responses did not differ significantly between groups (no group effect; *p* = 0.58). Figure 1 shows the observed rectal adaptation to isobaric distension. Increasing the pressure of distension increased the recorded initial rectal volumes (Fig. 1A) and maximal rectal volumes (Fig. 1B) (pressure effect, p < 0.0001 for each variable). Compared with the HVs, the recorded initial rectal volumes (rectal compliance) were significantly lower in the patients with SB (Fig. 1A, group effect, p = 0.01; interaction group pressure, p = 0.04). Global compliance was lower in patients with SB (SB: 7.5 [5.0–9.0], HV: 11.0 [8.2–13.5]; p = 0.001). An increasing pressure of distension decreased rectal tone (maximal volume–initial volume) (Fig. 1C) up to the threshold of 21 mmHg (pressure effect, p < 0.0001) in both groups. Compared with the HVs, rectal tone was significantly higher in the patients with SB up to the threshold of 21 mmHg (interaction group pressure; p = 0.001) (Fig. 1C). An increasing rectal pressure significantly increased rectal perception scores (pressure effect, p < 0.0001) (Supplementary material 4). Rectal perception tended to be decreased in patients with SB (group effect = 0.08). Anal response, rectal adaptation and rectal perception were comparable in the 3 patients treated with anticholinergics.

### Function and morphology of the intestinal epithelial barrier (IEB)

**Figure 2:**
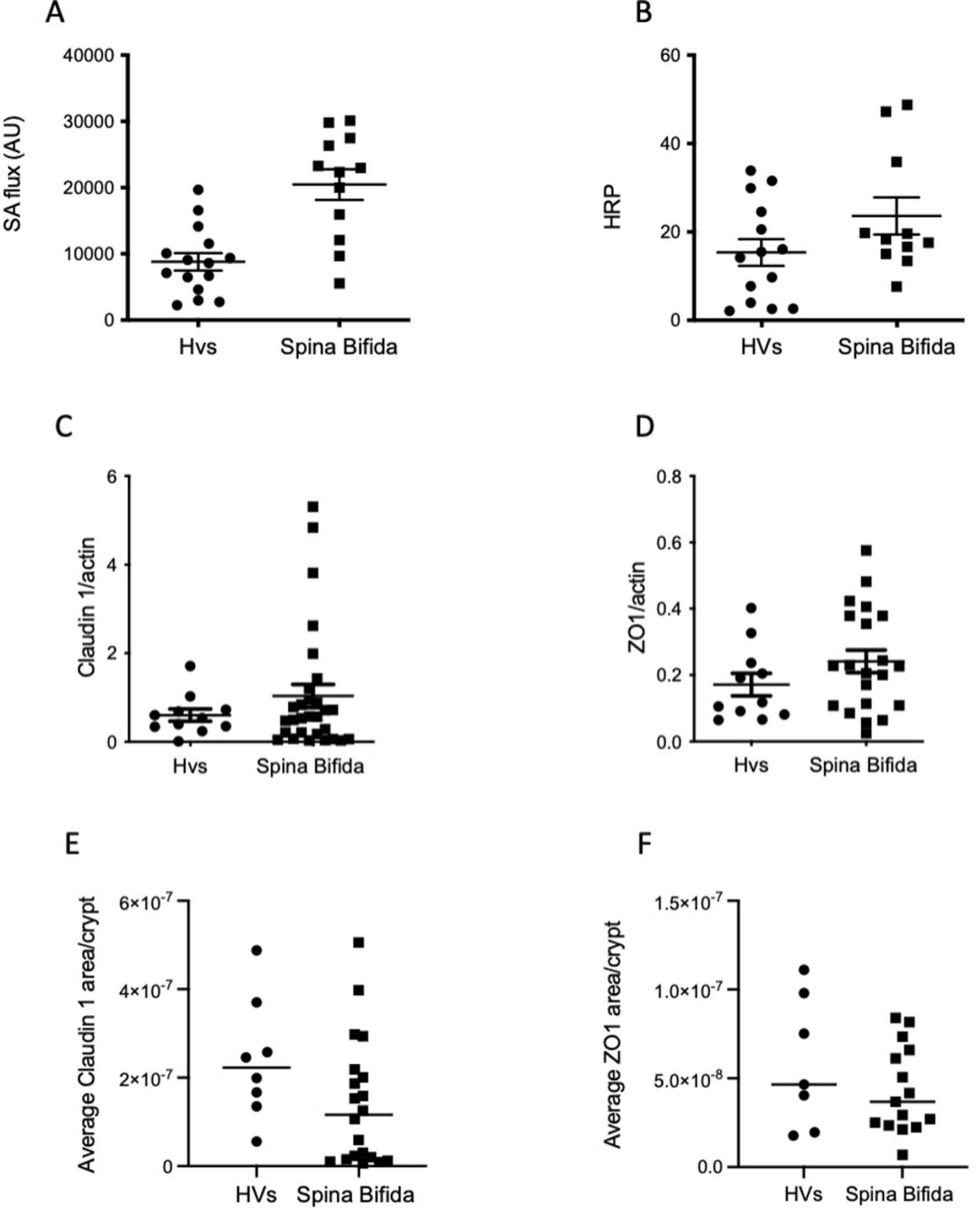
(**A**) and (**B**) Comparison of para- and transcellular permeability in patients with Spina Bifida and healthy volunteers (Hvs). (**A**) The paracellular permeability was significantly increased in patients with SB (p = 0008). (**B**) For the evaluation of transcellular permeability, no significant changes were observed between the two groups (p = 0.16). (**C**,**D**) The expression levels of claudin 1 and ZO1 assessed in Western Blot were comparable between the two groups. (**D**) The average claudin 1 area/crypt tended to be lower in patients with SB (p = 0.08). (**E**) The average ZO1 area /crypt were comparable between the two groups.

In the first set of experiments performed on biopsies, we evaluated whether IEB integrity was functionally altered in patients with SB compared to HVs (Fig. 2A,B). Paracellular permeability was significantly increased by 57% in patients with SB (p = 0008). Paracellular permeability was negatively and significantly correlated with rectal compliance (r = − 0.563, p = 0.002) (Table 3). The transcellular permeability was comparable between the two groups (p = 0.16). Transcellular permeability was not correlated with paracellular permeability or rectal compliance (p = 0.97).

**Table 3:** Spearman’s correlation coefficients with paracellular permeability and other parameters.

	Rectal compliance	Occludin expression (western blot)	Claudin-1 expression (immunochemistry)	Collagen total area (S-FFOCT)	Collagen proportion area (sirius red staining)
Paracellular permeability	r = − 0.563 p = 0.002	r = 0.6153 p = 0.01	r = 0.3828 p = 0.09	r = − 0.371 p = 0.07	r = − 0.1768 p = 0.08

Based on these results, we next investigated whether functional changes in the IEB functions observed in patients with SB were associated with changes in tight junction protein expression. The expression levels of occludin, Claudin-1, ZO1 and cingulin in colonic biopsies from patients with SB and HVs were analysed using Western blotting. The expression levels were comparable between the two groups (Fig. 2C,D; Supplementary materials 5 and 6). Nevertheless, occludin expression was positively correlated with paracellular permeability (r = 0.6153, p = 0.01) (Table 3). Next, by performing immunohistochemical staining of mucosal sections, we showed that the average ZO1- and JAMA-stained areas per crypt were comparable between the two groups (p = 0.14 and p = 0.60) (Fig. 2F; Supplementary materials 5 and 6). In contrast, the average Claudin-1-stained area per crypt tended to be lower in patients with SB (p = 0.08) (Fig. 2E) and tended to be positively correlated with paracellular permeability (r = 0.38, p = 0.08) (Table 3). In addition, we also showed that the proportion of cells Ki67+ was comparable between the two groups (p = 0.29).

### Morphological remodelling of the mucosal barrier in patients with SB

As no major change in the expression of tight junction proteins was observed, we next aimed to determine whether changes in IEB functions were associated with mucosal morphological remodelling. First, using S-FFOCT, we imaged the entire mucosal surface of two biopsies from each patient. We analyzed the morphological parameters of crypts and surface epithelial lining using previously validated criteria^30^. We found that the following parameters were comparable between the two groups: number of crypts/samples, mean area of a crypt, total crypt density, total density of crypts (%), elliptic form of crypts (major axis on minor axis), mean circularity, and mean roundness (p = 0.86, p = 0.73, p = 0.32, p = 0.22, p = 0.09, p = 0.61, and p = 0.77, respectively) (Supplementary material 7).

**Figure 3:**
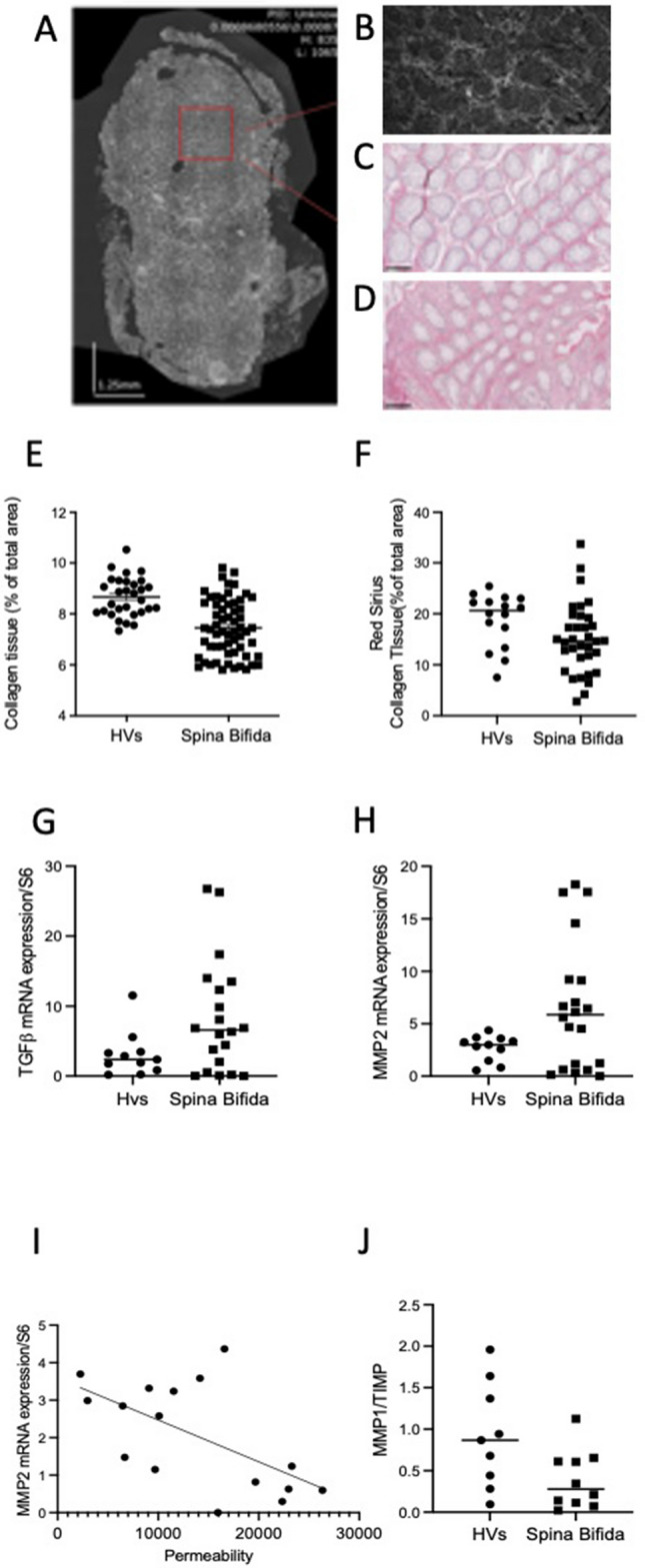
(**A**) A first macroscopic image of the whole biopsy was first obtained using a wide-field camera in order to screen for the regions of interest within the biopsy. Static FF-OCT images of the region of interest were then obtained at 2 depths. (**B**) Two images for each subject were assessed. (**C**) Image of biopsy of Hv stained with Sirius red. (**D**) Image of biopsy of SB stained with Sirius red. (**E**) The percentage of collagen per area were significantly lower in patients with SB (p = 0.0003). (**F**) The percentage of collagen per area after staining with red sirius was significantly lower in patients with SB (p = 0.0075). (**G**) The mRNA expression of TGF beta was significantly higher in patients with SB compared with Hvs (p = 0.0176). (**H**) The mRNA expression of MMP2 was significantly higher in patients with SB compared with Hvs (p = 0.0121) and tended to be correlated with the paracellular permeability (p = 0.0516) (**I**). (**J**) The TIMP1/MMP1 balance was significantly lower in patients with Spina Bifida (p = 0.0348).

Next, based on the optical properties of OCT that facilitate the identification of collagen-rich structures due to their high refractive index (Fig. 3A,B), we showed that collagen area and collagen density (evaluated by measuring the intensity of the signal) were significantly lower in patients with SB (p = 0.0003 and p = 0.0003) (Fig. 3E). We next analyzed Sirius red staining, which labels collagen structures, in mucosal tissue sections to confirm these findings. We showed that the area identified by Sirius red staining was significantly lower in patients with SB than in HVs (p = 0.008 (Fig. 3C,D,F). We showed that these 2 parameters (collagen density in OCT and Sirius red staining) tended to be negatively correlated with paracellular permeability (r = − 0.37, p = 0.07 and r = − 0.18, p = 0.08, respectively) (Table 2).

We next aimed to assess whether changes in collagen expression were associated with changes in the expression of molecules involved in its regulation, such as TGFβ1, MMP1, MMP2 and TIMP. The TGFβ1 and MMP2 mRNAs were expressed at significantly higher levels in patients with SB than in HVs (p = 0.02 and p = 0.01, respectively) (Fig. 3G,H). In addition, MMP2 mRNA expression tended to be correlated with paracellular permeability (p = 0.05) (Fig. 3I). TIMP1 and MMP1 mRNA expression levels were comparable between the two groups (p = 0.17 and p = 0.77, respectively). However, the MMP1/TIMP1 balance was significantly lower in patients with SB (p = 0.03) (Fig. 3J).

### Inflammation of the intestinal epithelial mucosa

We next aimed to determine whether functional changes in the IEB were associated with changes in colonic inflammatory mediators in patients with SB. Significantly higher TNFα mRNA expression, but not IL6, IL8, and IFNγ mRNA expression, was observed in patients with SB than in HVs (Supplementary material 8). Furthermore, the GDNF mRNA was expressed at significantly higher levels in patients with SB than in HVs (p = 0.008) (Supplementary material 8). GDNF mRNA expression was correlated with TNFα expression (p = 0.0001; r = 0.9001).

### Changes in microbial derived metabolites

**Figure 4:**
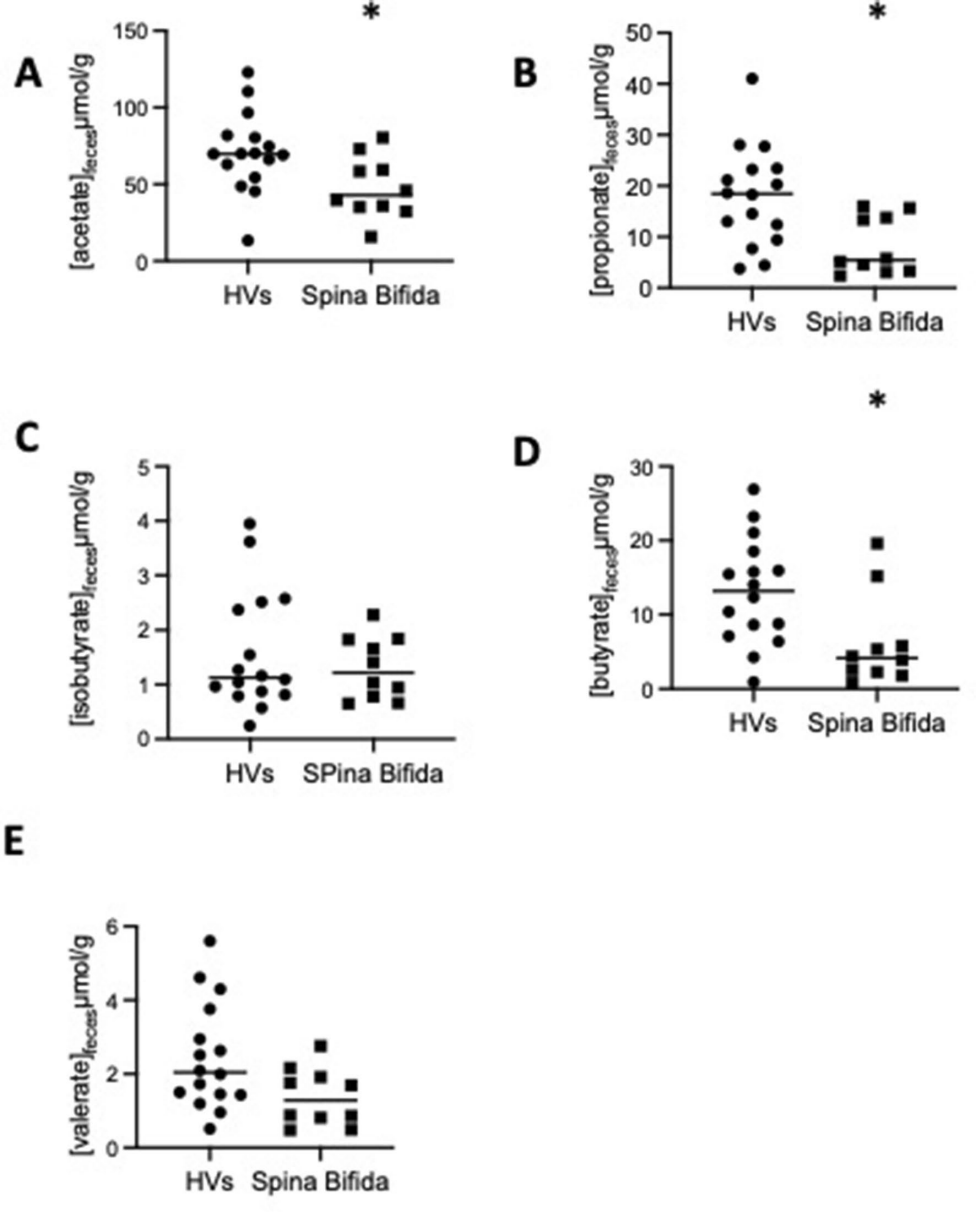
The expression levels of acetate, propionate and butyrate in fecal samples were significantly lower by 33, 54 and 53% in patients with SB compared with HVs (p = 0.02, p = 0.01, p = 0.02, respectively).

Finally, we aimed to determine whether changes in gut and barrier functions were associated with changes in key bacterial metabolites known to regulate barrier and motor functions, such as SCFAs and BAs. Significantly reduced levels of acetate, propionate and butyrate of 33, 54 and 53%, respectively, were detected in faecal samples from patients with SB than in samples from HVs (p = 0.02, p = 0.01, and p = 0.02, respectively) (Fig. 4). Concerning bile acid concentrations, the levels of CA, DCA, UDCA, and LCA were similar in HVs and patients with SB. As expected, glycol- and tauro-conjugated BAs or free HDCA were not detected in faecal samples. The concentrations of BAs or SCFAs were not correlated with paracellular permeability.

## Discussion

This study highlights that anorectal disorders in patients with SB involve not only anorectal dysmotility but also intestinal epithelial barrier dysfunction. Indeed, compared to HVs, patients with SB exhibited a conserved anal resting pressure but alterations in anal contraction characterized by (1) an increase in RAIR percentage of relaxation amplitude and duration and (2) a decrease in rectal compliance and an increase in rectal tone. Furthermore, patients with SB had increased ex vivo rectal paracellular but not transcellular permeability that was significantly negatively correlated with rectal compliance compared to HVs. Interestingly, an increase in Claudin-1 immunoreactivity in colonic epithelial cells was reported in patients with SB compared to HVs. However, no changes in the expression of other key tight junction proteins assessed using WB were observed in patients with SB compared to HVs. Furthermore, a significant reduction in the density of connective tissue was reported in patients with SB compared to HVs. Interestingly, the connective tissue density was negatively correlated with paracellular permeability. Furthermore, the TGFβ1, TNFα and GDNF mRNAs were expressed at higher levels in patients with SB. Finally, significantly reduced levels of the SCFAs acetate, butyrate and propionate were detected in patients with SB compared to HVs. Overall, our study reveals that abnormalities in rectal function in patients with SB are associated with abnormalities in rectal epithelial barrier functions and occur concomitantly with major changes in the concentrations of bacteria-derived SCFAs.

To our knowledge, our study is the first to perform a global functional and molecular assessment of anorectal functions in patients with SB, i.e., barrier and motility functions. One of the strengths of our work is based on the constitution and study of a group of HVs matched for age and sex with patients with SB who underwent the same assessments and sample collection methods under the same conditions. Furthermore, validated scales^23,25,26,31,32^, clinical exams, anorectal manometry with an electronic barostat, colonic biopsies and faecal samples were analysed in the two groups. Notably, the strengths of the study might be partially limited by the fact that the recruitment of patients with SB through a national referral centre might have resulted in selection bias. The analyzed group was, however, representative of the referral centre population since the prevalence rates of severe FI (50 vs. 60%), constipation (78 vs. 85%) and severe neurological bowel dysfunction (33 vs. 42%) were comparable in the present sample and in the whole cohort^3^.

The major findings of this study are that patients with SB exhibited both changes in rectal compliance and rectal tone. Regarding rectal compliance, these data are consistent with studies evaluating rectal compliance^10^ in neurological patients. Rectal compliance reflects the active adaptation of the rectal wall to pressure variations and is controlled by the autonomic nervous system. The parasympathetic system is thought to excite the bowel, and if damaged, such as in patients with SB, it would be expected to lead to a reduction in rectal compliance. Reduced rectal compliance may be an additional factor contributing to FI in patients with SB, impairing the ability of the rectum to act as a capacitance vessel. The association of an increased RAIR amplitude and reduced rectal compliance has yet to be described as a factor contributing to FI^8^. The increased RAIR amplitude has important functional implications, as it will reduce anal resting pressure and may predispose some patients with SB to FI. Our work also showed that the RAIR percentage of relaxation amplitude and duration were significantly greater in patients with SB, as described in patients with spinal cord injury^10,33,34^. Additionally, the association of an increased RAIR amplitude and reduced rectal compliance described in our study might partially explain FI in patients with SB. Moreover, parasympathetic modulation of the RAIR has been reported^10^, but the RAIR is presumed to be purely subject to enteric modulation, which may suggest damage to the enteric nervous system.

Importantly, we showed that patients with SB exhibited changes in rectal tone. Rectal tone is thought to reflect parietal viscoelastic properties. To our knowledge, only one study^35^ investigated variations in rectal tone in neurological patients, and no studies have evaluated rectal tone in patients with SB. Using rectal impedance planimetry, the authors showed^35^ that rectal tone was reduced in patients with conal or cauda equina lesions and increased in patients with supraconal spinal cord lesions. These data are consistent with our results and suggest that rectal tone is stimulated by the sacral spinal cord. The control of rectal tone is very complex and little is known about this mechanism. The data associated with changes in rectal tone are limited because the only method to reliably study it is to use the rectal barostat.

Another major finding of this work is that we reported an increase in paracellular permeability associated with an increase in inflammatory cytokine levels. However, the molecular mechanisms responsible for the increased permeability observed in patients with SB remain to be determined but do not seem to involve changes in the expression TJ proteins studied here, such as ZO-1 or occludin. However, one cannot exclude that altered localization of TJ such as ZO-1 can occur in SB. Particular, cellular/subcellular localization could have also contributed to altered permeability. This alter point deserves however further studies. Alternatively, the involvement of other candidate TJ proteins of the claudin, as claudin 2 or ZO family^36^, as well as MLCK which has been reported to regulate permeability^37^ remain to be explored in future study, as limited amount of proteins from the biopsies hampered us to study these candidate in the current study.

Our study revealed an ‘inflammatory state’ of the mucosa in patients with SB, as characterized by an increase in the expression of TNFα and MMP2 but surprisingly not of other key cytokines such as IL-8 or IFN-γ. Although this study was assessed at the mRNA levels, it remains to be determined whether mucosal cytokine production is indeed enhanced in SB patients as compared to HVs. Increased permeability of the IEB is associated with inflammation of the gut in several digestive disorders, such as inflammatory bowel diseases^17,18^ and irritable bowel syndrome (IBS)^13,14^, and even in neurological diseases, such as Parkinson's disease^19^. The increased expression of TNFα and MMP2^38^ might explain in part the increased permeability observed in patients with SB. In particular, TNFα has been shown in various models to increase paracellular permeability via the regulation of TJ proteins or by inducing intestinal epithelial cells death^39^. In addition to changes in permeability, we also reported morphological changes in the mucosa that were characterized by reduced connective tissue levels. This loss of connective tissue is also observed in mucosal inflammatory states^40^ and reduced connective tissue could hamper epithelial cells restitution following injury^41^. Concomitantly, this mucosal ‘pro-inflammatory’ response, i.e., increased permeability and expression of TNFα, MMP2, is also associated with molecular responses that might reflect a mucosal adaptative and pro-reparative response. Indeed, first, we observed an increase in expression of TGFβ1 and Claudin-1 mRNAs, which have been shown to contribute to the restoration of gut barrier integrity^21^. Interestingly, increased expression of TGFβ1 was associated with increased expression of Claudin-1^42^. Another mediator that has been shown to exert protective/reparative effect on IEB is GDNF whose mRNA expression was found to be increased and positively correlated with TNFα in SB patients as compared to control. In particular, GDNF has been shown to prevent TNFα-induced increases in paracellular permeability^43^ in animal models of inflammation in part by prevented TNFα induced cells death in intestinal epithelial cells^44^. Therefore, increased GDNF expression might limit increased permeability in patients with SB. Finally, a reduced MMP1/TIMP1 balance in patients with SB might also contribute to limiting the collagen degradation reported in our study and thereby favour repair. Altogether, our data suggest that functional changes in permeability observed in patients with SB might be due to mucosal inflammation and/or defect in intestinal barrier function such as repair processes.

Finally, changes in rectal barrier functions and inflammation observed in patients with SB have been shown to be associated with changes in SCFA levels, as characterized by a reduction in faecal SCFA concentrations, particularly butyrate levels. These changes in SCFAs might reflect altered composition of the gut microbiota in SB patients. Among reduced expression of key SCFAs levels observed in our study is butyrate. It is therefore tempting to speculate that reduced levels of butyrate could contribute to the barrier dysfunctions and inflammatory remodeling of the gut mucosa observed in patients with SB. Indeed, butyrate has been shown to reduce paracellular permeability and/or protect against barrier dysfunction induced by inflammatory mediators^45–47^. In addition, butyrate also exerts direct immunomodulatory effects, and in particular, butyrate has been shown to reduce TNFα production in the human mucosa subjected to inflammatory stress^48^. Finally, butyrate has also been shown to regulate motor functions by increasing colonic transit^49^. Therefore, it remains to be determined whether reduced butyrate levels may contribute to the motor and barrier dysfunctions observed in patients with SB. Interestingly, an improvement in digestive disorders following colonic irrigation was recently shown to be associated with changes in the gut microbiota and, in particular, an increase in the proportion of *Roseburia*^50^, which is a major butyrate-producing bacterium. Altogether, further studies are needed to determine whether changes in motility induced by spina cord lesions contribute to decreased expression of butyrate-producing bacteria that subsequently enhance constipation-associated motor and barrier dysfunctions in patients with SB. Nevertheless, the role of butyrate as a potential therapeutic target to improve gut functions in patients with SB remains a topic of interest.

## Conclusions

Our findings provide the first evidence that anorectal and epithelial barrier functions are concomitantly altered in adult patients with SB. Furthermore, our findings revealed the presence of a mucosal inflammatory response and altered faecal contents of SCFAs in patients with SB. Future therapies aimed at restoring barrier functions and/or the gut microbiota might represent new promising therapeutic approaches for patients with SB.

## Supplementary Information

Supplementary Information.
