# Supplementary material for: Altered epithelial barrier functions in the colon of patients with spina bifida
Source: Sci Rep. 2022 May 3;12:7196. doi: 10.1038/s41598-022-11289-3 (PMC9065040; doi:10.1038/s41598-022-11289-3)
Supplement: Supplementary file 1 — Supplementary Information. [file 41598_2022_11289_MOESM1_ESM.docx]

**Supplementary material 1**

1. **Description of *Ex vivo* assessment of colonic para- and transcellular permeability**

Three biopsies from each HV and patient with SB were mounted in Ussing chambers (Physiologic Instruments, San Diego, USA), exposing a surface of 0.011 cm². Each side of the tissues were bathed with 3 ml of F12-supplemented Dulbecco’s modified Eagle medium (Invitrogen, France) containing 0.1% (v/v) foetal bovine serum, 200 mM glutamine and 45 g/L NaHCO3. The medium was continuously oxygenated and maintained at 37 °C under gas flow (95% O2/5% CO2). After a 30 min baseline period, 200 μL of apical medium were replaced with 200 μL of media containing 1 mg/mL fluorescein-5,6-sulfonic acid (molecular weight: 400 Da) (Life Technologies) at a final concentration of 0.1 mg/mL to assess paracellular permeability and 10 mg/mL horseradish peroxidase (HRP) (Sigma ‒Aldrich, Saint-Quentin-Fallavier, France) at a final concentration of 0.375 mg/mL to measure transcellular permeability. The fluorescence level of basolateral aliquots of 150 μl, reflecting paracellular passage from the luminal surface, was measured every 30 min over a 3-hour period using a fluorimeter (Varioskan®, Thermo Fisher Scientific, Cillebon sur Yvette, France). The enzymatic activity of HRP, reflecting transcellular passage from the apical surface, was measured every hour over a 3-hour period using an enzymatic activity assay with 3,3’,5,5’-tetramethylbenzidine reagent (BD Bioscience, Le Pont de Claix, France). Paracellular and transcellular permeabilities were determined by calculating the slope of fluorescence or absorbance intensity over time in the three biopsies from each HV or patient with SB.

1. **Description of Western Blot analysis and details of antibodies used :**

Samples of mucosa from patients with SB and HVs were lysed with RIPA lysis buffer (Millipore, USA) containing sodium orthovanadate, a phosphatase inhibitor cocktail II (Roche, France) and a protease inhibitor cocktail (Complete®; Roche, France) in tubes containing 0.6–0.8 mm ceramic beads using a Precellys 24 tissue homogenizer (Bertin Technologies, France) followed by sonication with a Vibracell 75 186 device (Sonics, Newton CT, USA). Total protein concentrations were quantified using a bicinchoninic acid protein assay kit (Thermo Fisher, USA). Equal amounts of lysate (12 μl containing 12 μg of proteins) were separated on Invitrogen NuPage Novex 4-12% Bis-Tris MidiGels® and NuPage Novex 3-8% Bis-Tris MidiGels® (to reveal ZO1) using Bis-Tris running buffer before electrophoretic transfer to nitrocellulose membranes with an iBlot 2® Dry Blotting System (Invitrogen, USA). Membranes were duplicated. Membranes were placed in a 10% acetic acid bath for 10 minutes, washed with Tris-buffered saline (TBS) (150 mM NaCl, 15 mM Tris, and 4.6 mM Tris Base, pH 7.4) and blocked overnight at 4 °C with TBS containing 5% nonfat dry milk. Membranes were cut. Membranes were incubated overnight at 4 °C with primary antibodies **(Table above).** Bound antibodies were detected with horseradish peroxidase-conjugated anti-rabbit antibodies (1:5000; Thermo Fisher Scientific, USA) and visualized using enhanced chemiluminescent detection (ECL Prime, France). The relevant immunoreactive bands were quantified with laser-scanning densitometry and analysed with Image Lab™ Software (Bio-Rad, France). The levels of ZO-1, Occludin, Claudin and Cingulin were normalized to the amount of beta-actin and reported as a percentage of the average of controls.

Details of antibodies used for Western blotting.

| **Antibody** | **Host** | **Catalogue number, source** | **Dilution** |
| --- | --- | --- | --- |
| ZO-1 | Rabbit | 402-200, Life | 1:200 |
| Occludin | Rabbit | ab167161, Abcam | 1:50 000 |
| Claudin 1 | Rabbit | ab180158, Abcam | 1:200 |
| JAMA | Rabbit | A 302891, Bethyl | 1:1000 |
| Cingulin | Rabbit | Sc 66831, Santa Cruz | 1:500 |
| Horseradish peroxidase | Anti Rabbit | Thermo Fisher Scientific | 1:5000 |

1. **Immunohistochemical and staining studies**

Two mucosal biopsies from all subjects were fixed with formalin, embedded in paraffin and routinely stained with haematoxylin & eosin to exclude the presence of microscopic colitis. The paraffin-embedded tissues were cut with a microtome, mounted on a SuperFrost+ slide and dried at 56 °C for 4 hours. Sections were dewaxed with EZ Prep Roche® for 8 minutes at 75 °C; antigen unmasking was performed with CC1 ® (Tris Ph8) buffer. Immunohistochemistry was performed using the DISCOVERY ULTRA system (Roche ®, France). Sections were incubated with primary antibodies detailed above (**Table**). Immunohistochemical staining for occludin and cingulin was not possible (nonspecific labelling). After additional standard Ventana™ processing and counterstaining with haematoxylin and bluing solution, sections were scanned with a high-resolution Hamamatsu Nanozoomer 2.O RS scanner. Digital analysis software (Halo, Indicalabs) was trained to recognize colon crypts and ZO-1/Claudin-1/JAMA staining. Colon crypt areas were determined using the manual selection tool to outline the crypts. Each delimited area of interest in each section was analyzed with the IHC area algorithm modified as necessary for ZO-1, Claudin-1, JAMA and quantified. The methods of dewaxing, marking and analysis were the same for the analysis of Ki67 proportion. A section of the formalin-fixed, paraffin-embedded mucosal biopsy was cut and stained with Sirius red to determine the collagen proportion area.

Details of antibodies used for immunochemical studies.

| **Antibody** | **Catalogue number, source** | **Dilution** |
| --- | --- | --- |
| ZO-1 | Invitrogen 339100-clone 1A12 | 1:200 |
| Claudin 1 | ab180158, Abcam | 1:100 |
| JAMA | A 302891, Bethyl | 1:100 |

1. **FFOCT imaging**

Microdissection of 2 biopsies was performed as previously described (33). Each whole-mount preparation was fixed with 4% paraformaldehyde for 3 hours, washed 3 times with 1X PBS (phosphate-buffered saline) and then stored at 4 °C in PBS/NaN_3_ for further analyses. We used full-field optical coherence tomography (FFOCT), as described recently (34), to further analyse the morphology of the epithelial barrier. The samples were fixed and analysed using the static mode of FFOCT. A macroscopic image of the whole biopsy was obtained using the wide-field camera mode to screen for regions of interest. S-FFOCT images of the selected region were acquired at multiple depths. ImageJ software (1.42q, National Institutes of Health, USA) was used for the quantitative assessment. Two images of samples from each subject were assessed quantitatively by an independent investigator (RO) who measured the following parameters: number of crypts/sample, mean area of a crypt, total crypt density, total density of crypts (%), elliptic form of crypts (major axis on minor axis), mean circularity, mean roundness, collagen total area, and collagen % area. These parameters are those collected in confocal endomicroscopy imaging studies of the epithelial barrier (35). A manual method of contouring the crypts was performed. This method was reproducible between 2 investigators (RO and CB). For collagen analysis, the Imaging J software threshold adjustment was used to select the percentage of grey level on the slice corresponding to collagen in the image and to quantify it. The image was formatted as "8-bit", the threshold was adjusted to 120 (the best ratio to select connective tissue)

1. **Supplementary material 3: Description of Quantitative Polymerase Chain Reaction (qPCR) and details of primers used**

Total RNA was extracted from the mucosal samples crushed in 600 µl of RA1 with a Precellys 24 tissue homogenizer (Bertin Technologies, France) in tubes containing 0.6–0.8 mm ceramic beads using nucleospin RNA II (Macherey-Nagel, France). After RNA extraction, potential genomic DNA contamination was removed by treatment with Turbo™ DNase (Ambion Inc., USA), and RNA was quantified using a Nanodrop 2000 spectrophotometer (Nanodrop Technologies, Wilmington, USA). The reverse transcriptase reaction was performed with 1 µg of total RNA that had previously been denatured at 72 °C for 3 minutes using the Super Script III Reverse Transcriptase System kit (Life Technologies, France) in a Thermal Cycler 2720 (Applied Biosystems) (25 °C for 5 minutes, 50 °C for 55 minutes, 70 °C for 15 minutes). The cDNAs were then diluted to a final concentration of 4 ng eq RNA/µl. The sense and anti-sense oligonucleotide primers (TGFβ1, IL8, IL6, TNFα, IFNγ, GDNF, TIMP, MMP1, and MMP2) used in this study are shown in **the table above**. Amplifications were performed in duplicate using a StepOne Plus (Life Technologies, France) detection system with Fast SYBR Green (Life Technologies) master mix. A standard curve was generated with serial dilutions of control cDNAs by plotting the relative amounts of these dilutions against the corresponding Ct (cycle threshold) values. The expression level of each gene was calculated from these standard curves using StepOnePlus® software. The expression of the S6 ribosomal protein was used as a reference. For each sample, the ratio between the relative amount of each specific transcript and S6 was calculated to normalize possible variations in mRNA levels. Samples were tested in duplicate, and the mean values were used for quantification using the 2^-ddCT method, as previously described.

Details of primers used for quantitative polymerase chain reaction.

| Primer | Gene | Sequence (5' - 3') |
| --- | --- | --- |
| h-TGFβ1 | Transforming growth factor beta | GTCACCGGAGTTGTGCGGCA  CTCGGCGGCCGGTAGTGAAC |
| h-IL8 | Interleukin beta 8 | CTGGCCGTGGCTCTCTTGG  ATTTCTGTGTTGGCGCAGTGTG |
| h-IL6 | Interleukin 6 | CAATGAGGAGACTTGCCTGGTGAA  TGTGGTTGGGTCAGGGGTGGTT |
| h-TNFα | Tumour necrosis factor alpha | CCCGAGTGACAAGCCTGTAG  TGAGGTACAGGCCCTCTGAT |
| h-IFNγ | Interferon gamma | CCAGAGCATCCAAAAGAGTGTGGAG  GCTGGCGACAGTTCAGCCATCA |
| TIMP-1 | Tissue inhibitors of metalloproteinase -1 | CAAGATGTATAAAGGGTTCCAAGC  TCCATCCTGCAGTATTCCAC |
| MMP-1 | Matrix metalloproteinase -1 | GTCTCGGAGGAGATGCTCACGCC  AAGTTCATGAGTTGCAG |
| MMP-2 | Matrix metalloproteinase-2 | AAGTATGGCTTCTGCCTGA  ATTTGTTGCCCAGGAAAGTG |
| GDNF | Glial cell line-derived neurotrophic factor | GCTGCCCGCCGGTAAGA  TGGTGGCTTGAATAAAATCCATGAC |

1. **Bacterial derived metabolite analysis**

Subjects provided fresh faecal samples collected in the morning on the day of the colonoscopy. The faecal samples were then aliquoted and snap frozen in liquid nitrogen at the time of collection and stored at −80 °C until analysis. SCFAs were measured using gas chromatography–mass spectrometry (GC–MS). A stock solution of SCFA metabolites (Sigma–Aldrich) was prepared and serially diluted to obtain ten calibration solutions (i.e., 60-30,000 µmol/L for acetate, 6-3,000 µmol/L for propionate, and 0.6-300 mmol/L for butyrate/isobutyrate/valerate). A working solution of internal standards was prepared in 0.15 M sodium hydroxide (NaOH) to obtain the following final concentrations: 75 mmol/L *d_3_*-acetate, 3.8 mmol/L *d_5_*-propionate, 2.5 mmol/L *^13^C*-butyrate and 0.5 mmol/L *d_9_*-valerate (Sigma–Aldrich). Faecal samples were weighed (~50 mg) and dissolved in 200 µL of 0.15 M NaOH (Sigma–Aldrich). Twenty microlitres of the internal standard solution were added to the samples and calibration solutions. Each sample was then acidified with 5 µL of 37% hydroxide chloride (Sigma–Aldrich) and extracted with 1.7 mL of diethyl ether (Biosolve, France). Samples were stirred gently for 1 h and then centrifuged for 2 min (5,000 rpm, 4 °C). The organic layers were transferred to 1.5 ml glass vials, and SCFAs were derivatized with 20 µL of tert-butyldimethylsilyl imidazole (Sigma–Aldrich). Samples were incubated for 30 min at 60 °C before analysis. Samples were finally analysed using GC–MS (Model 7890A-5975C; Agilent Technologies, France) equipped with a 30 m × 0.25 mm × 0.25 µm capillary column (HP1-MS; Agilent Technologies). The temperature program started at 50 °C for 1 min, ramped to 90 °C at 5 °C/min and then increased to 300 °C at 70 °C/min. Selected ion monitoring mode was used to measure SCFA concentrations with ions at a mass-to charge ratio (*m/z*) of 117 (acetate), 120 (*d_3_*-acetate), 131 (propionate), 136 (*d_5_*-propionate), 145 (butyrate and isobutyrate), 146 (*^13^C*-butyrate), 159 (valerate) and 168 (*d_9_*-valerate).

Bile acids (BAs) were analysed in faecal samples using liquid chromatography-tandem mass spectrometry (LC–MS/MS). All solvents were LC–MS grade and purchased from Biosolve. Standard compounds were obtained from Sigma‒Aldrich. Faecal samples were weighed (~50 mg) and dissolved in ~500 µL (i.e., 10 mL for 1 g, dilution 1/10) of water. A pool of reference standard solutions (cholic acid (CA), chenodeoxycholic acid (CDCA), deoxycholic acid (DCA), ursodeoxycholic acid (UDCA), hyodeoxycholic acid (HDCA) and lithocholic acid (LCA) in their free and tauro- and glycol-conjugated forms) was prepared and serially diluted in water to obtain 7 standard solutions ranging from 0.1-100 µg/mL (equivalent to 0.1-100 µg/g). Ten microlitres (10 μL) of exogenous internal standards at 100 μg/mL in 50% methanol (*d_4_*-CA, *d_4_*-glyco-CDCA, and *d_4_*-tauro-UDCA) were added to 500 μL of standard solutions and faecal samples. All samples were then mixed in an ultrasonic bath for 15 min. After complete homogenization, samples were acidified with 25 µL of 37% hydroxide chloride and then extracted with 1.2 mL of ethyl acetate (Biosolve, France). Samples were centrifuged for 10 min (5,000 rpm; 4 °C). Supernatants were collected and dried under a gentle stream of nitrogen. Dried samples were finally dissolved in 600 µL of 50% acetonitrile and injected (10 µL) into the LC–MS/MS. LC–MS/MS analyses were performed using a Xevo^®^ TQD mass spectrometer with an electrospray interface and an Acquity H-Class^®^ UPLC^TM^ device (Waters Corporation, Milford, MA, USA). Samples (5 μL) were injected into a CORTECS UPLC C18 column (1.6 μm; 2.1 × 100 mm, Waters Corporation) held at 60 °C, and compounds were separated with a linear gradient of mobile phase B (50% acetonitrile, 50% isopropanol, 0.1% formic acid, and 10 mM ammonium formate) in mobile phase A (5% acetonitrile, 0.1% formic acid, and 10 mM ammonium formate) at a flow rate of 400 μL/min. Mobile phase B was maintained at a constant volume of 10% for 1 min linearly increased from 10% to 60% for 9 min, linearly increased from 60% to 95% for 1 min, held constant at 95% for 1 min, returned to the initial condition (10%) over 1 min, and held constant for 1 min before the next injection. Targeted compounds were then detected using the mass spectrometer with the electrospray interface operating in negative ion mode (capillary voltage, 2 kV; desolvation gas (N_2_) flow rate and temperature, 1000 L/h and 400 °C, respectively; source temperature, 150 °C). Multiple reaction monitoring mode was applied for MS/MS detection, as detailed above:

**Multiple reaction monitoring (MRM) parameters used for LC–MS/MS detection of bile acids.**

| **Compound** | **MRM transitions (*m/z*)** | **Cone/Collision (V)** |
| --- | --- | --- |
| CA | 407.3 → 343.3 | 70/34 |
| DCA | 391.3 → 343.3 | 70/34 |
| CDCA, UDCA, HDCA | 391.3 → 391.3 | 70/15 |
| LCA | 375.3 → 375.3 | 70/15 |
| Glyco-CA | 464.3 → 74.0 | 70/40 |
| Glyco-DCA, glyco-CDCA, glyco-UDCA, glyco-HDCA | 448.3 → 74.0 | 70/40 |
| Glyco-LCA | 432.3 → 74.0 | 70/40 |
| Tauro-CA | 514.4 → 79.9 | 70/70 |
| Tauro-DCA, tauro-CDCA, tauro-UDCA, tauro-HDCA | 598.4 → 79.9 | 70/70 |
| Tauro-LCA | 482.4 → 79.9 | 70/70 |
| *d_4_*-CA | 411.3 → 347.3 | 70/34 |
| *d_4_*-glyco-CDCA | 452.4 → 74.0 | 70/40 |
| *d_4_*-tauro-UDCA | 502.4 → 79.9 | 70/70 |

**Supplementary material 2 : Anorectal manometry**

|  | ***Patients with spina bifida***  ***N=36*** | |  | ***Healthy volunteers***  ***N=16*** |  |
| --- | --- | --- | --- | --- | --- |
| ***Variable*** |  | *N (%) or* median [IQR] |  | *N (%) or* median [IQR] | ***P value*** |
| **Anorectal manometry** |  |  |  |  |  |
| Amplitude of anal contraction (mmHg) |  | 0 [0.0-25.5] |  | 53 [37.5-76.5] | *<0.0001* |
| Mean resting pressure |  | 50.5 [39.8-69.8] |  | 55.5 [48.0-64.0] | *0.5065* |
|  |  | N=29 |  | N=16 |  |
| Rectal perception |  |  |  |  |  |
| Threshold perception volume (ml) |  | 30 [20.0-40.0] |  | 25 [15.0-30.0] | *0.2102* |
| Defecatory urge volume (ml) |  | 105 [60.0-25.5] |  | 115 [90.0-135.0] | *0.3960* |
| Maximum tolerable volume (ml) |  | 195 [145.0-290.0] |  | 230 [185.0-255.0] | *0.4050* |
| RAIR (presence) |  | 32 (94.1) |  | 16 (100) | *1.0000* |
| Amplitude RAIR (% at 50 ml) |  | 96.5 [71.4-100.0]  18.8 [14.7-25.8] |  | 66.0 [43.5-82.5]  11.0 [7.0-15.5] | *0.0009*  *0.0002* |
| Duration of RAIR (seconds) |  |  |  |  |  |

**Abbreviations**: SD=standard deviation; RAIR=rectoanal inhibitory reflex

**Supplementary material 3**: Residual pressures (RAIR) recorded at upper part of the anal canal as a function of induced rectal pressures. Results were comparable when recorded at the lower part of the canal anal. Rectal distensions induced a pressure-dependent relaxation of anal canal (pressure effect ; *p* < 0.0001). Anal responses did not differ significantly between groups (no group effect ; *p* =0.5744).

✺ Patients with Spina Bifida; O Healthy volunteers


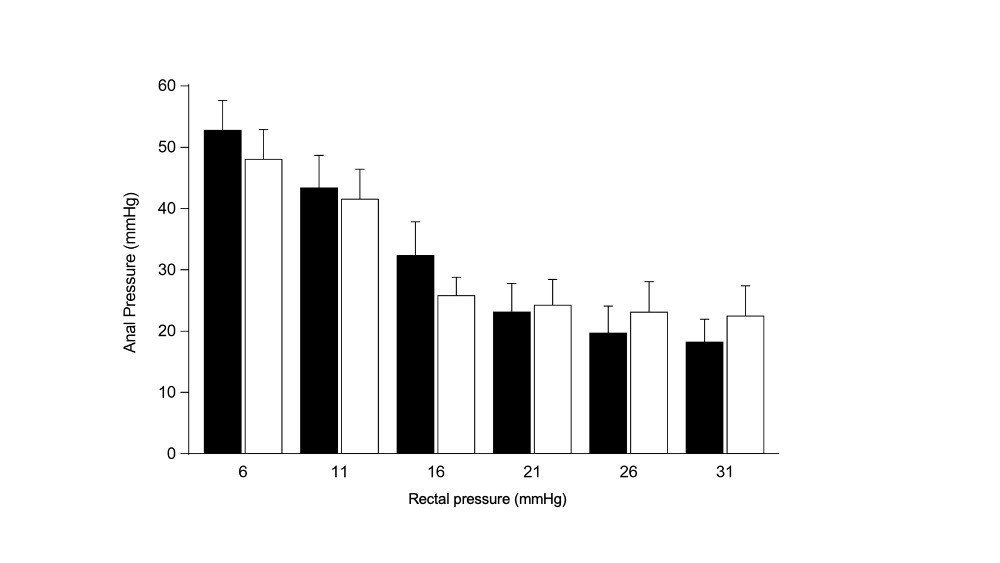


**Supplementary material 4** : Rectal perception observed in response to isobaric distension. Increasing rectal pressure significantly increased rectal perception scores (pressure effect, p<0.0001). Rectal perception tended to be decreased in patients with SB (group effect= 0.0761).

✺ Patients with Spina Bifida; O Healthy volunteers


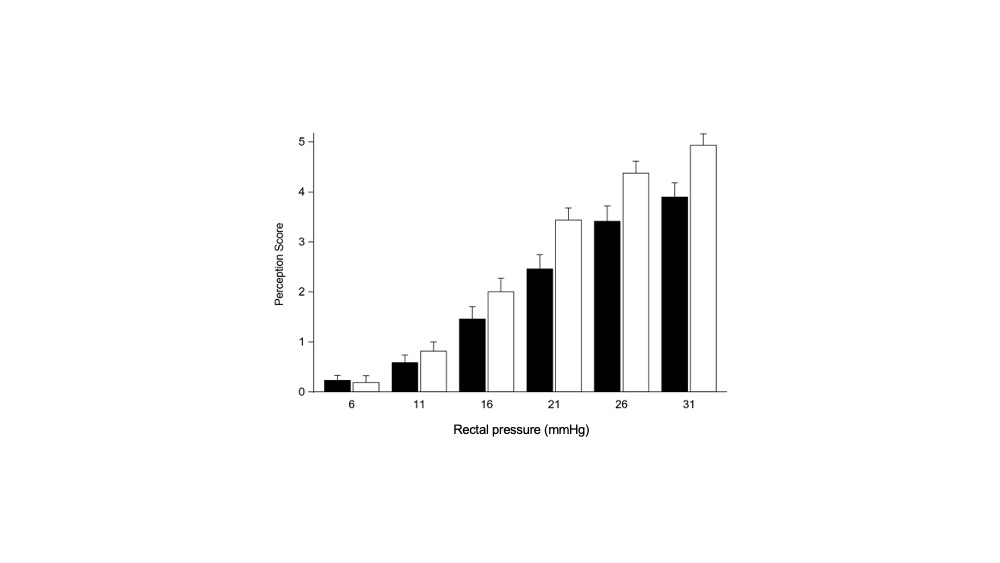


**Supplementary material 5**

Representative images of Claudin 1 expression in Western Blot (and actin) (A) and immunohistochemistry (C) and ZO1 expression in Western Blot (and actin) (B) and immunohistochemistry (D) in both healthy volunteers and patients with Spina Bifida.


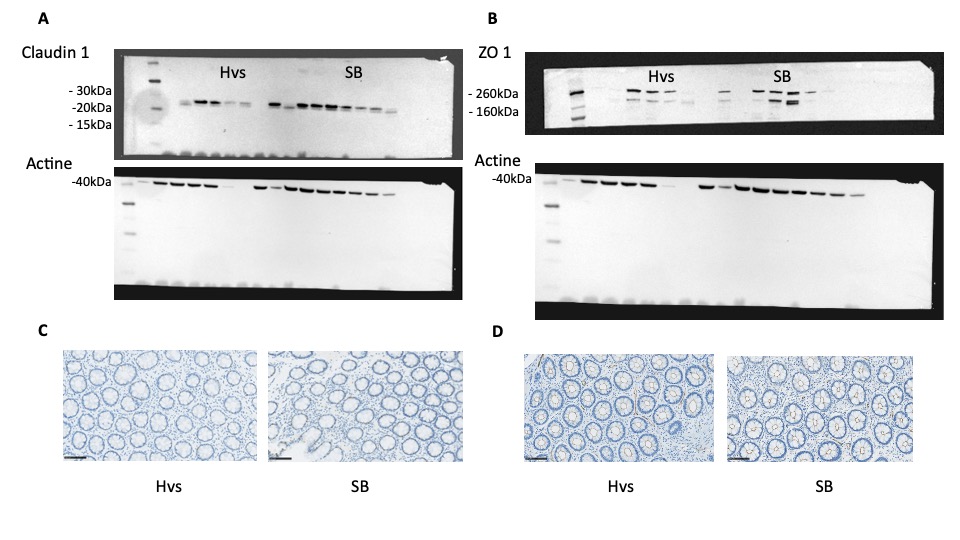


**Supplementary material 6:**

The expression levels of occludin and cingulin assessed in Western Blot were comparable between the two groups. The average JAMA area/crypt and cingulin area/crypt assessed in immunohistochemistry were comparable between the two groups.


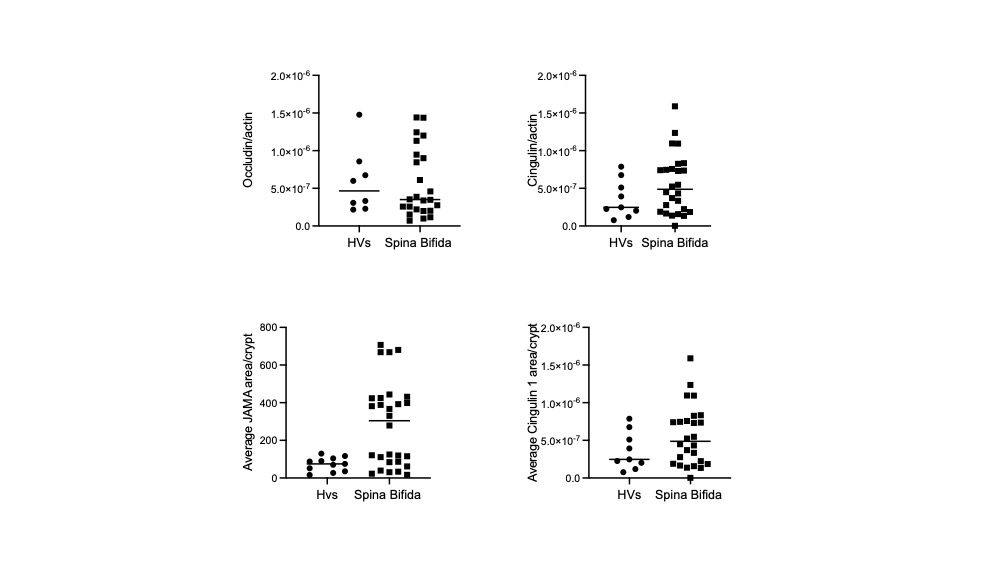


**Supplementary material 7:** Number of crypts/samples, mean area of a crypt, total crypt density, total density of crypts (%), elliptic form of crypts (major axis on minor axis), mean circularity, and mean roundness (p=0.8588, p=0.7310, p=0.3251, p=0.2159, p=0.0900, p=0.6102, and p=0.7669, respectively) were comparable between the two groups
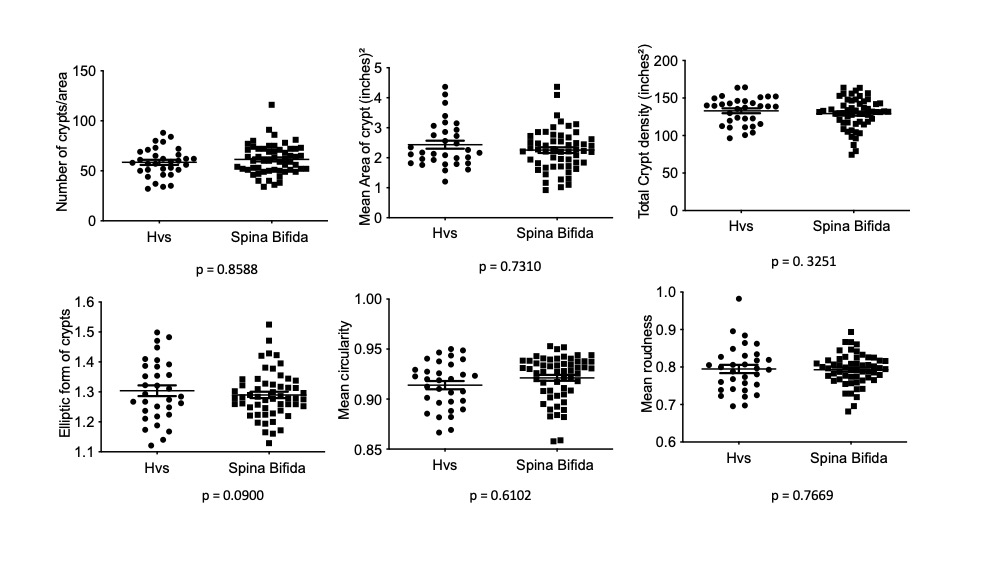


**Supplementary material 8:** The mRNA expression levels of IL6 (A), IL8 (B), and IFNγ (D) were comparable between the two groups (p= 0.1494, p= 0.6188 and p=0.0912)

The mRNA expression level of TNFα (C) was higher in patients with Spina Bifida (p=0.005)

The mRNA expression level of GDNF (E) was higher in patients with Spina Bifida (p=0.005)

**
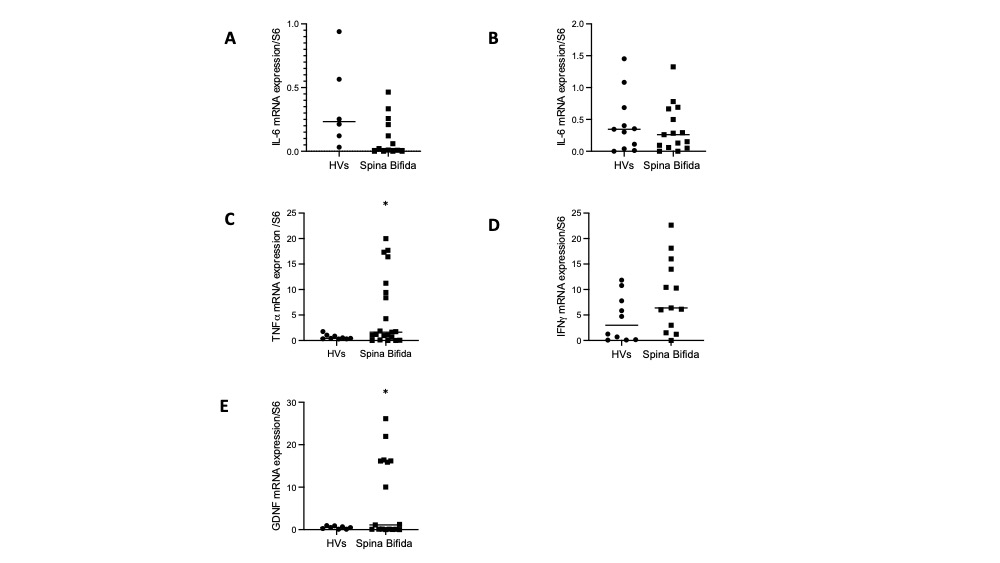
**
